# Effect of UV-C on the physiology and biochemical profile of fresh *Piper nigrum* berries

**DOI:** 10.1016/j.postharvbio.2017.11.007

**Published:** 2018-02

**Authors:** Emma R. Collings, M. Carmen Alamar Gavidia, Katherine Cools, Sally Redfern, Leon A. Terry

**Affiliations:** a Plant Science Laboratory, Cranfield University, Bedfordshire, MK43 OAL, UK; b Unilever R&D Colworth, Sharnbrook, Bedfordshire, MK44 1JA, UK

**Keywords:** Black pepper, Real-time respiration rate, Ethylene, Piperine, Essential oils

## Abstract

Application of UV-C has been shown to enhance the biochemical profile of various plant materials. This could be used to increase biochemical load, reducing the amount of material required but still impart equivalent flavour. As spices, such as black pepper (*Piper nigrum* L.), are typically dried to low moisture content to create a stable product for transportation and storage, little work has explored the use of modern postharvest treatments to enhance flavour. In this work, fresh *P. nigrum* berries were exposed to four UV-C doses (0, 1, 5 and 15 kJ m^−2^) and subsequently stored at 5 °C for *ca*. 4 weeks. Two separate experiments (early and late season) were conducted across one season. Replicate *P. nigrum* berry clusters were stored separately within continuously ventilated 13 L boxes. Real-time respiration rate (*ex situ*), ethylene production, fruit colour and water potential were measured at regular intervals during storage. In addition, piperine and essential oils were assessed using a simple newly developed method which enabled both compound groups to be simultaneously extracted and subsequently quantified. UV-C was found to cause significant changes in colour (from green to brown) whilst also altering the biochemical composition (piperine and essential oils), which was influenced by UV-C dose and berry maturity. Low to medium UV-C doses could potentially enhance flavour compounds in black pepper enabling processors to create products with higher biochemical load.

## Highlights

• UV-C caused significant changes in colour (from green to brown).
• A low UV-C dose significantly increased piperine and essential oil content.
• A newly developed simultaneous extraction method is discussed.

## 1 Introduction

Dried black pepper berries, known as peppercorns, are the most widely traded spice in the world, representing 20% of all spice imports (Parthasarathy et al., 2008). Commercial black pepper (*Piper nigrum* L.), which is produced from mature berry fruit, is typically sun dried to reduce water content to below 10% and thus ensure product stability (Ravindran, 2000). Piperine, which imparts pungency, is the most abundant alkaloid in black pepper and has been suggested to have anti-carcinogenic effects and enhance the bioavailability of certain drugs/biochemical compounds (Srinivasan, 2007). In addition, black pepper essentials oils have been reported to possess antioxidant and antibacterial properties, anti-oncogenic potential (Misharina et al., 2009, Butt et al., 2013, Zarringhalam et al., 2013) and have also been shown to improve swallowing reflexes in elderly dysphagic people (Ebihara et al., 2010).

Application of ultraviolet-C light (UV-C), which occupies the non-ionising region of the electromagnetic spectrum between 200 and 280 nm, has the potential to increase the biochemical content in fresh produce. Low doses of potentially harmful agents, such as UV-C, are reported to induce beneficial stress responses in plant products (termed ‘plant hormesis’) (Terry and Joyce, 2004). Plant products with increased biochemical content induced through hormesis have been proposed to confer benefits when consumed, referred to as xenohormesis (Hooper et al., 2010).

Advances in postharvest technology and storage could offer alternative processing methods for spices such as black pepper, which could enhance biochemical load. However, studies on the impact of UV-C on the biochemical content and moisture content of fresh *P. nigrum* berries are not available. In this work, two studies were performed using different levels of UV-C applied to imported fresh *P. nigrum* berries to determine the impact on physiology and biochemical profile (*viz.* piperine and essential oils).

## 2 Materials and methods

In 2014, two consignments of fresh *P. nigrum* berry stalks termed rachides (cv. Sri Lanka from the same season) were sourced from Somsak Suksum based in Thailand (Thamai District, Chanthaburi province). Samples were harvested on 18th January (consignment 1 – early season) and 23rd June (consignment 2 − late season), and received by Cranfield University on 27th January and 30th June, respectively. After harvest, both consignments were transported for 4 h by non-refrigerated truck before being re-packaging into a polystyrene box lined with perforated plastic and gel ice packs.

### 2.1 Experimental design and sampling

UV-C irradiation (254 nm) was applied at different doses (kJ m^−2^) to consignment 1 (0 [control], 1 [UV-C _1_] and 15 kJ m^−2^ [UV-C_15_]) and consignment 2 (0 [control], 1 [UV-C_1_], 5 [UV-C_5_] and 15 kJ m^−2^ [UV-C_15_]) using a custom-built rack, which was placed inside an enclosed fume hood for protection against exposure. Irradiance, emitted by ten lamps (30 W, TUV-TL-D 254 nm, Phillips, Surrey, UK) mounted onto the rack, was measured using an optical radiometer (Multisense-100, Ultraviolet Products, Cambs., UK). Samples for irradiance were placed directly underneath the bulbs. To prevent overheating and excessive moisture loss during UV-C treatment, samples were placed onto trays, containing cool water (*ca.* 5 °C), with samples positioned within gridded sections. For high UV-C doses (15 kJ m^2^), samples were exposed to an average intensity (fluence rate) of 8.3 W m^2^ for 30 min which was split into 4 × 7.5 min intervals. In contrast, samples treated with a medium (5 kJ m^2^) and low (1 kJ m^2^) UV-C dose were exposed for a total of 10 and 2 min, which were split into 4 × 2.5 min and 2 × 1 min intervals, respectively. After each UV-C treatment interval, surface temperature was assessed using a non-contact infrared thermometer (Fisher Scientific, Traceable^®^). Prior to starting the next UV-C dose interval, water in the tray was removed and replaced with fresh water, and samples were randomly repositioned to account for possible variations in UV-C intensity between the centre and outer edges. During all UV-C dose applications, surface temperature did not exceed 17 °C.

After treatment, each of the three batches (containing *n* = 32 rachides) per UV-C dose, were placed into separate 13 L Sealfresh™ polypropylene boxes (31 × 31 × 15 cm) to give three replicates per treatment. A subset of rachides was withheld from UV-C exposure to act as control; these were placed directly into the 13 L boxes.

All 13 L storage boxes were sealed with vacuum grease and constantly flushed with air (1.8 L min^−1^) from inside the cool room (at 5 °C) using a blower manifold (custom built and supplied by Air Equipment, Beds., UK) to prevent accumulation of carbon dioxide (CO_2_) and ethylene inside the boxes. Air entering the boxes was bubbled through deionised water to maintain a constant high RH.

### 2.2 Physiological measurements

Real-time respiration rate was measured *ex situ* using 3 L jars (four rachides per jar [*n* = 3 reps per treatment]) and analysed using a Sable Respirometry System (Model 1.3.8 Pro, Sable Systems International, Las Vegas, USA) as described in Collings et al. (2013) with slight modifications. Using ‘push mode’, each sample jar was measured for 2 min, to achieve a stable reading, while a baseline (empty jar) measurement (for 1 min) was recorded in-between.

Ethylene measurements were performed using an ETD-300 Ethylene Detector with a CAT-1 catalyser and VC-1 valve control box (Sensor Sense, Netherlands) (Elmi et al., 2013). Each sample, consisting of four rachides per sample box (*n* = 3 replicates per treatment), were placed into cuvettes (635 mL) which had a continuous flow (4 L h^−1^) of hydrocarbon-free air flushing through. A stable baseline measurement, obtained using an empty cuvette, was recorded at the beginning and end of each run to allow values to be adjusted by removing the baseline. Sample cuvettes were measured for *ca.* 6 to 10 min to allow a stable measurement to be recorded. To ensure the air in each cuvette had been completely exchanged after closing, thus removing potentially hydrocarbon contaminated air, cuvettes were flushed for up to 30 min.

Objective colour (lightness (L*), chroma (C*) and hue angle (h°) were measured using a Minolta CR-400 colorimeter with an 8 mm light aperture and DP-400 data processor (Minolta Co. Ltd., Japan). The mean was calculated based on three readings; these readings were taken from the proximal, centre and distal end of the rachis (*n* = 4 rachides per replicate).

Water potential was initially recorded as water activity (a_w_) using an AquaLab (Series 3, Version 1.7) dew point meter. A five-point calibration curve was produced using known concentrations of glycerol to produce specific water activity readings (between 1 and 0.8). This allowed the% deviation between actual a_w_ value and meter reading to be calculated and sample readings were adjusted accordingly. Water activity was measured using a pooled sample containing 5 berries from each rachis (*n* = 4) per replicate.

Moisture content was determined for each treatment per outturn (*n* = 3 replicates) using fresh (non-freeze dried) ground powder (*ca.* 400 mg). Each sample was placed in an oven and dried at 60 °C for 16 h. After removal, samples were placed into a desiccator, containing silica gel beads, for 30 min to allow the vials to cool under dry conditions. The difference in vial weight (plus sample) before and after drying was used to determine% moisture. Samples were placed back into the oven for a further 1 h and the weight recorded to ensure a constant weight had been achieved.

### 2.3 Biochemical analysis

A newly developed simultaneous extraction of piperine and essential oils in *P. nigrum* berries was performed on *ca*. 500 mg of fresh weight powder. Snap-frozen pepper was prepared for biochemical analysis by randomly selecting five berries per rachis (*n* = 4 rachides per replicate) and grinding into fine powder using an automated mortar grinder under liquid nitrogen (Retsch RM 200, Germany). Samples were weighed into a 15 mL plastic centrifuge tube (which had been pre-cooled) and transferred immediately to −40 °C. To maintain a low temperature upon removal of the cap, the tubes were placed immediately in ice and 1 mL of pre-cooled (*ca.* 2 °C) acetone was added. Samples were vortexed and placed in an ultra-sonic bath at room temperature for 15 min. After extraction, samples were re-vortexed, centrifuged at 2830*g*-force for 5 min and then placed at −40 °C for 5 min. Once cooled, the acetone was decanted into a pre-cooled 2 mL plastic tube and placed back into −40 °C. The extraction process was repeated on the same sample and the two extracts combined. Samples were filtered, using a pre-cooled syringe and 0.2 μm PTFE filter, into a pre-cooled amber HPLC vial.

For piperine quantification using an Agilent 1200 series HPLC system with DAD, extracts required a 50:1 dilution before injection (10 μL) into an Agilent ZORBAX Eclipse XDB-C18 column (4.6 mm x 250 mm, 5 μm particle size, Part no. 990967-902) with an OPTI-GUARD 1 mm Guard Column, C18 (Part no. 10-02-00007). The mobile phase consisted of A: 30% acetonitrile and 0.5% formic acid, B: 100% acetonitrile. Separation was achieved using a gradient with a linear increase/decrease of solvent B: 0–8 0%, 15 min; 80-0%, 5 min; plus a post run of 0%, 5 min at a flow rate of 0.05 L h^−1^ and a column temperature of 25 °C. Compounds were detected using a photodiode array detector set to a wavelength of 220 and 340 nm and quantified against an authentic standard purchased from Sigma Aldrich (UK). Piperine values were expressed in a DW basis following adjustment using moisture content data.

Essential oil analysis was performed using an Agilent gas chromatograph Model 6890N network GC system, Agilent Technologies (Agilent, Berks., UK) equipped with a flame ionisation detector (FID). Extracts (non-diluted) were injected (1 μL) into a Zebron ZB-Wax Plus (Part no. 7HM-G013-11) capillary column (30 m long, 0.32 mm i.d., 0.25 μm film thickness; Phenominex, Macclesfield, Cheshire). Injector and detector temperature were maintained at 250 °C and Helium was used as the carrier gas with a split ratio of 70:1. Total analysis time was 46 min per sample. The temperature program was set as follows: 60 °C for 11 min, 4 °C a min^−1^ to 120 °C which was held for 5 min, 6 °C min^−1^ to 180 °C held for 5 min. Peak identification was achieved by comparison with known standards (α-pinene, β-pinene, 3-carene, α-phellandrene, terpinene, limonene, sabinene and β-caryophyllene) purchased from Sigma Aldrich (Dorset, UK). As with piperine, values for essential oil content are expressed on a DW basis following adjustment using moisture content data.

### 2.4 Statistical analysis

Statistical analyses were carried out using Statistica for Windows version 10, 64-bit (Statsoft Tulsa, OK 74104, USA). Analysis of variance (ANOVA) was used to identify any significant differences (*P* < 0.05) between treatments. LSD bars are displayed on all graphs (including non-significant (*P* > 0.05)).

## 3 Results

### 3.1 Maturity and colour

The first consignment (Exp. 1) of *P. nigrum* rachides received from Thailand 9 d after harvest, comprised of berries with diameters *ca.* 1.27-fold larger compared to consignment 2 (Exp. 2), which arrived 7 d from harvest. In addition, consignment 1 contained a number of mature red berries. However, despite the presence of red berries within consignment 1, comparison of objective colour measurements upon initial receipt at CU showed only significant differences in lightness, but not chroma or hue angle.

During storage at 5 °C, values for lightness, chroma and hue angle significantly decreased in both experiments as pepper berries changed from green to black. This colour change was more rapid in pepper exposed to 15 kJ m^2^ UV-C with overall mean values being significantly lower during storage compared to the control and UV-C_1_ treated pepper (Table 1). Lightness and hue angle values for UV-C_15_ in Exp. 2 were not significantly different to UV-C_5_ pepper.

**Table 1:** Effect of treatment (UV-C_1_, UV-C_5_ and UV-C_15_; 1, 5 and 15 kJ m^−2^, respectively) on overall mean values for lightness, chroma and hue angle from Exp. 1 and 2 observed in fresh P. nigrum berries cv. Sri Lanka during storage at 5 °C. Significant differences (P < 0.05) are denoted by different letters within a column. Data represent means (n = 3).

Treatment	Exp. 1			Exp. 2		
	Lightness	Chroma	Hue angle	Lightness	Chroma	Hue angle
Baseline	32.13^d^	25.68^c^	115.45^a^	32.76^d^	25.66^e^	117.87^c^
Control	29.43^c^	21.35^a^	111.35^a^	27.85^c^	17.65^d^	102.86^b^
UV-C_1_	28.68^b^	20.69^a^	109.92^a^	26.90^b^	16.67^c^	101.02^b^
UV-C_5_	–	–	–	25.86^a^	15.31^b^	97.28^a^
UV-C_15_	26.07^a^	15.89^b^	103.81^b^	25.26^a^	14.25^a^	96.38^a^

### 3.2 Respiration rate and ethylene production

**Fig. 1:**
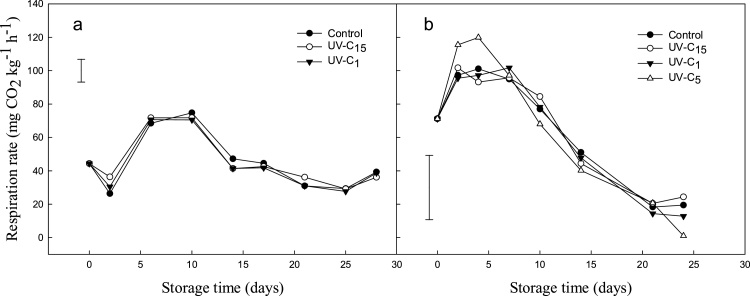
Effect of UV-C in Exp. 1 (a) (UV-C_1_ and UV-C_15_; 1 and 15 kJ m^−2^, respectively) and Exp. 2 (b) (UV-C_1_, UV-C_5_ and UV-C_15_; 1, 5 and 15 kJ m^−2^, respectively) on respiration rate (mg kg^−1^ h^−1^ at RT) in fresh P. nigrum berries cv. Sri Lanka during 28 and 24 d storage at 5 °C, respectively. Data represent means (n = 3). Bars represent LSD (P < 0.05).

For both experiments, *ex situ* respiration rate was found to range between *ca.* 20 to 115 mg CO_2_ kg^−1^ h^−1^, and in line with that reported by others on climacteric and non-climacteric bush berry fruit (UC Davis, 2013). A climacteric-like increase was observed in fresh *P. nigrum* after day two and at day zero in Exp. 1 and 2, respectively (Fig. 1). However, in Exp. 2, the climacteric peak appeared to have started prior to receiving the pepper, with values starting at *ca.* 77 mg CO_2_ kg^−1^ h^−1^ and falling below <20 mg CO_2_ kg^−1^ h^−1^. In contrast, respiration rate in Exp. 1 was initially recorded at *ca.* 30 mg kg^−1^ h^−1^ before increasing up to 77 mg kg^−1^ h^−1^ after two days of storage. Values were maintained up to day ten before decreasing to initial values observed at the beginning of storage.

Ethylene production followed a similar pattern to respiration rate where a climacteric increase occurred in Exp. 1 after day zero with values reaching *ca.* 0.94 μg kg^−1^ h^−1^ before decreasing to <0.12 μg kg^−1^ h^−1^ (detection limit). In contrast, ethylene production in Exp. 2 started at an initial high value of *ca.* 12 μg kg^−1^ h^−1^ and continued to decrease during storage with values falling below the detection limit (data not shown). The climacteric peak for ethylene production in Exp. 2 was 10-fold higher compared to that observed in Exp. 1.

### 3.3 Moisture content and water potential

In both experiments, control samples were found to have overall significantly lower moisture content compared to the UV-C treated berries (Table 2). In addition, pepper treated with 1 kJ m^−2^ UV-C during Exp. 1 were found to have an overall significantly higher moisture content (72.2%) compared to UV-C _15_ (70.6%).

**Table 2:** Effect of UV-C dose in Exp. 1 (UV-C_1_ and UV-C_15_; 1 and 15 kJ m^−2^, respectively) and Exp. 2 (UV-C_1_, UV-C_5_ and UV-C_15_; 1, 5 and 15 kJ m^−2^, respectively) on moisture content (%) and water potential (MPa) in fresh P. nigrum berries cv. Sri Lanka during 28 and 24 d storage at 5 °C, respectively. LSD (P < 0.05) values are shown in bold and significant differences are denoted by different letters (within a row).

Exp.	Measurement	*Baseline*	*Control*	*UV-C_1_*	*UV-C_5_*	*UV-C_15_*	*LSD (P <* *0.05)*
1	Moisture (%)	70.1^abc^	67.8^a^	72.2^c^	–	70.6^b^	**1.34**
2	Moisture (%)	83.8^a^	82.09^b^	83.5^a^	83.4^a^	83.2^a^	**0.53**
2	Water potential (MPa)	−7.54^ab^	−6.71^a^	−5.43^c^	−5.82^bc^	−6.00^abc^	**0.89**

Water potential values remained similar during storage. However, there was a significant treatment effect where the control (-6.71 MPa) had significantly lower water potential compared to UV-C_1_ (-5.43 MPa) and UV-C _5_ (-5.82 MPa) (Table 2). This treatment effect was consistent for moisture content where the control also had significantly lower values compared to UV-C treated pepper. However, UV-C_15_ (-6.00 MPa) was not significantly different to either control or the other two UV-C treatments (1 and 5 kJ m^−2^ UV-C).

### 3.4 Piperine and essential oils

During both storage experiments, piperine concentration was found to range between 40 and 80 g kg^−1^ (only data from Exp. 1 shown Table 3), which is in line with that reported by others (Suresh et al., 2007). In Exp. 1, an overall treatment effect was observed where both UV-C_15_ and UV-C_1_ treated berries had significantly higher values compared to control. However, no differences in piperine concentration were detected as a result of the two different doses (15 and 1 kJ m^−2^). In the repeat experiment (Exp. 2.), no treatment effect was found. In Exp. 1, a positive correlation between piperine and moisture content was observed (r^2^ = 0.66) during storage.

**Table 3:** Effect of UV-C (UV-C_1_ and UV-C_15_; 1 and 15 kJ m^−2^, respectively) in Exp. 1 on overall piperine and essential oil content (g kg^−1^) in fresh P. nigrum berries cv. Sri Lanka during storage at 5 °C. Data represent means (n = 3). LSD (P <0.05) values are shown in bold and significant differences are denoted by a different letter (within a row).

	Experiment 1 g kg^−1^				
Compound	*Baseline*	*Control*	*UV-C_1_*	*UV-C_15_*	*LSD (P <* *0.05)*
Piperine	48.59^ab^	48.14^b^	53.73^a^	51.73^a^	**3.34**
α-pinene	1.56^a^	1.84^a^	2.07^b^	1.84^a^	**0.35**
β-pinene	3.84^a^	4.25^a^	4.78^b^	4.34^a^	**0.71**
α-phellandrene	0.56^a^	0.61^a^	0.69^b^	0.62^a^	**0.10**
Limonene	6.04^a^	6.33^a^	7.07^b^	6.44^a^	**0.97**
Sabinene	0.63^a^	0.66^a^	0.69^a^	0.61^a^	**0.13**
β-caryophyllene	11.88^ab^	11.83^a^	13.06^b^	11.44^a^	**1.75**

Six essential oils (*viz.* α-pinene, β-pinene, α-phellandrene, limonene, sabinene β-caryophyllene) were identified and quantified in pepper samples cv. Sri Lanka from both experiments using GC-FID. As observed with piperine content, exposure to 1 kJ m^−2^ UV-C in Exp. 1 was found to have an overall treatment effect where mean values for five of the essential oils (*viz.* α-pinene, β-pinene, limonene and β-caryophyllene) were significantly higher compared to control and UV-C _15_ (Table 3).

## 4 Discussion

### 4.1 Effect of UV-C on metabolic rate

Earlier work by Latifah and co-workers (1998) indicated that *P. nigrum* was a climacteric fruit where berries (cv. Semongok Emas) at breaker stage were found to have 3-fold higher respiration rate compared to green mature and immature berries. In addition, respiration values were reported to range between 0.88 and 2.83 mg kg^−1^ h^−1^ at ambient temperature (28 °C). However, a complete climacteric rise and fall in respiration rate was not recorded as measurements were discontinued after 4 d at 28 °C (due to fungus growth). Further studies on the climacteric behaviour of *P. nigrum* are absent. In this study, climacteric peaks in respiration rate and ethylene production were recorded during both Exp 1 and 2. However, values were found to be up to 60-fold higher than that recorded by Latifah et al. (1998). Discrepancies in measurements obtained by Latifah et al. (1998) and that recorded for pepper cv. Sri Lanka could be associated with differences in cultivar or more likely due to methodology (*viz*. temperature and sample volume: flow rate).

The climacteric peak observed during Exp. 2 appeared to have already started prior to performing respiration rate measurements. It is unclear why this occurred in Exp. 2, as berries were more immature and experienced shorter transportation/storage duration (seven days) compared to berries received in Exp. 2 (nine days). One possible explanation is that the two batches of pepper were exposed to different temperatures during transportation, which may have triggered an earlier climacteric in Exp. 2 samples.

In both experiments, no significant differences in respiration rate or ethylene production were observed between treatments suggesting that UV-C dose had no influence on metabolic rate. A similar result was also observed on another vine fruit where UV-C treatments did not have any effect on respiration rate in red table grapes (Freitas et al., 2015).

### 4.2 P. nigrum berry maturity

Despite the observed climacteric increases in respiration and ethylene production, both batches of pepper berries failed to fully ripen during storage. During the experiment, pepper berries did not undergo the typical colour change from green to red which is indicative of ripening. During the ripening process, piperine and essential oil content in fresh green pepper are also reported to decrease (Jansz et al., 1983). However, in this study, no decrease in biochemical content (*viz*. piperine and essential oils), which coincided with the lack of ripening colour change, was observed. Failure to ripen may have been attributed to maturity. There is very little research on the potential for postharvest ripening behaviour of pepper (*P. nigrum*). Consequently, additional work would be required to fully understand the physiological and biochemical changes that occur during the different stages of maturity and ripening.

The efficacy of the applied UV-C treatments between the two experiments varied considerably. One reason for this could be differences in maturity between the two batches, as batch 1 was observed to have larger red berries (indication of maturity/ripeness) compared to batch 2 which appeared more immature (small and pale green). Objective colour measurements did not detect these differences which were perceived subjectively. These measurements were, however, limited to a small number of randomly selected pepper rachides. Future measurements on overall colour measurements across the entire batch could provide more accurate colour readings between batches.

### 4.3 Effect of UV-C on biochemical profile

In the majority of studies, essential oil content in *P. nigrum* is expressed as a% of the total essential oil content. However, as the essential oils reported in this work were extracted in acetone, as opposed to the traditional method of hydrodistillation, total oil content was not determined, and thus, values were instead reported as μg^−1^. The hydrodistilation method for extracting essential oils was not performed in this study due to small sample sizes. That said, implementing the newly proposed simultaneous extraction method for piperine and essential oils was found to be cost and time effective, as well as reproducible.

An increase in biochemical profile from UV-C exposure, as observed in Exp. 1, has previously been reported in other fresh produce such as tomato and broccoli (Liu et al., 2012, Costa et al., 2006). This could have been a stress response as previously hypothesised for grapevine berries cv. Pinot noir, where UV-C (2.98 kJ m^−2^) exposure was found to induce the expression of CH14D and TL3, which are two major defence related proteins (Colas et al., 2012). Furthermore, induced production of terpenoids has been reported in response to herbivorous damage (Cheng et al., 2007). In *P. nigrum* berries, two systems are involved in terpenoid synthesis and these include the mevalonic acid pathway (cytosol) and the non-mevalonic pathway (chloroplast). In this study, both terpenoid pathways (monterpenoids [α-pinene, β-pinene limonene and α-phellandrene] and sesquiterpenoids [β-caryophyllene] were similarly affected by exposure to UV-C as both were increased in UV-C_1_ pepper. However, the greatest increase was observed for β-caryophyllene, which has a woody-spicy, dry odour (Schulz et al., 2005).

### 4.4 Optimising UV-C dose

High UV-C doses (15 kJ m^−2^ in Exp.1) may have had a negative impact on biochemical profile as previously reported in blueberry fruit (Wang et al., 2009). In Maharaj et al. (2010) a UV-C hyper dose of 24.4 kJ m^2^ also significantly reduced the main carotenoid lycopene, in tomato fruit. In addition, abnormal browning was observed on the surface of these tomato fruit and it was hypothesised that this was associated with membrane damage resulting in phenolic oxidation via increased PPO activity (Maharaj et al., 2010). Surface browning was observed in pepper berries treated with the high UV-C dose of 15 kJ m^−2^.

### 4.5 Commercial benefits of UV-C application on final pepper quality

Experts involved in pepper grading assess product quality based on various criteria including size and colour of the peppercorn. High quality pepper products are regarded as being large, round and with a uniform black colour. In addition, high pungency and complex aroma (imparted by high essential oils) is also considered as a major factor in determining quality (Ravindran, 2000, Schulz et al., 2005). In this study, colour change was observed in UV-C treated pepper, which could potentially impact on the final colour quality of the product. It is postulated that UV-C caused membrane damage resulting in phenolic oxidation and also increases in moisture due to high water influx through the pericarp. As the surface temperature did not exceed 17 °C during the various UV-C treatments, it is unlikely that these physiological changes are an effect of temperature differences. The main phenolic compounds oxidised in pepper are reported to be 3,4-dihydroxy phenyl ethanol glycoside and 3,4-dihydroxy-6-(N-ethylamino)benzamide (Bandyopadhyay et al., 1990). More recent detailed studies on phenolic compounds in pepper are again lacking. Currently, blanching pepper in boiling water prior to drying is performed to achieve a uniform black colour (Ravindran, 2000). Uniform UV-C application could be achieved by placing threshed berries onto a conveyor belt-like system during treatment. In addition to providing the necessary colour change required for black pepper production, application of UV-C could also elicit alkaloid accumulation producing a consumer product with enhanced biochemical content.

## 5 Conclusions

Application of UV-C at low doses was found to enhance biochemical profiles of black pepper with implications that less material could be used but still impart equivalent flavour. Furthermore, a product with superior biochemical load could command higher prices and alleviate supply demands; thus, producing a more economically sustainable product with reduced impact on the environment. Implementing UV-C treatments into an industrial setting would be relatively straightforward since UV-C is already used commercially for various practices.
